# Case Report: Cardiac transplantation in a 76-year-old recipient: moving from anagraphic to biological age under a geriatric perspective

**DOI:** 10.3389/frtra.2025.1595938

**Published:** 2025-07-24

**Authors:** Lorenzo Giovannico, Federica Mazzone, Giuseppe Fischetti, Domenico Parigino, Luca Savino, Claudia Leo, Giuseppe Cristiano, Tommaso Acquaviva, Nicola Di Bari, Massimo Padalino, Tomaso Bottio

**Affiliations:** Cardiac Surgery Unit, Department of Precision and Regenerative Medicine and Ionian Area (DiMePRe-J), University of Bari Medical School, Bari, Italy

**Keywords:** heart transplantation, elderly patients, donor criteria, heart failure treatment, transplant eligibility

## Abstract

**Background/objectives:**

Heart transplantation remains the definitive treatment for end-stage heart failure. However, donor shortages and the increasing age of candidates present significant challenges. This report aims to highlight the feasibility and successful outcome of heart transplantation in an elderly patient, questioning traditional age-based eligibility criteria.

**Methods:**

A 76-year-old male with idiopathic dilated cardiomyopathy and severe heart failure underwent orthotopic heart transplantation. Preoperative assessments included right heart catheterization, echocardiography, and cardiac index evaluation. A suitable 66-year-old female donor was identified, and transplantation was performed using the bicaval technique. Postoperative outcomes were monitored through echocardiography and biopsy analysis.

**Results:**

The patient had an uneventful postoperative course, with extubation on day 1 and discharge on postoperative day 30. Follow-up at 14 months showed excellent clinical recovery, with an improved left ventricular ejection fraction (LVEF) of 58% and global longitudinal strain (GLS) of −20.8%. No signs of rejection were observed on biopsy.

**Conclusions:**

This case represents the oldest documented successful heart transplant recipient discharged home. The findings suggest that age alone should not be a limiting factor in transplantation eligibility. Expanding criteria to include well-selected elderly patients could help address the growing demand for donor hearts.

## Graphical Abstract

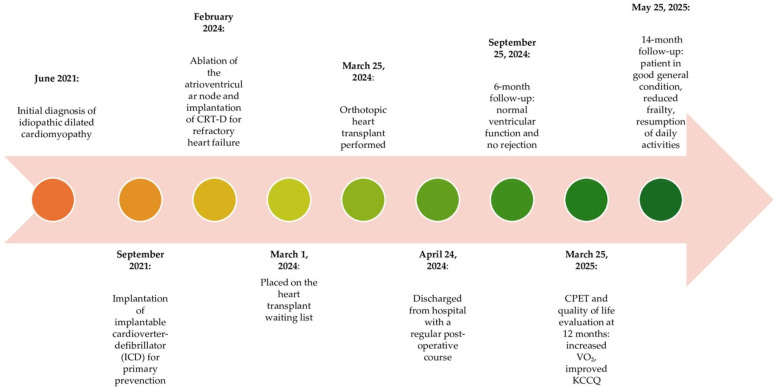

Timeline of the clinical course of a 76-year-old heart transplant recipient.

## Introduction

**Figure 1:**
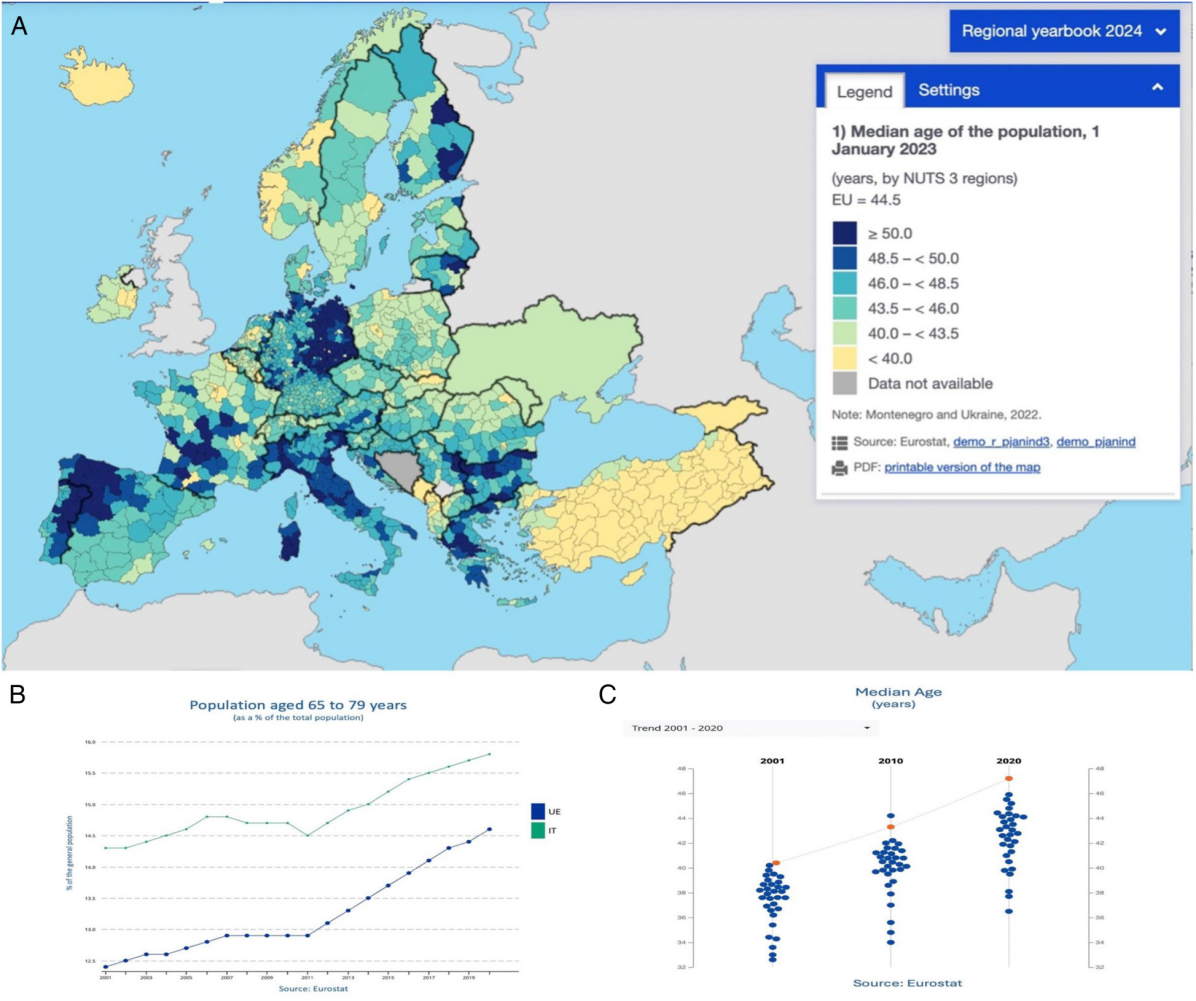
**(A)** Median age and aging population in Europe – 2023. This map displays the median age of the population across Europe as of January 1, 2023. Data is organized by NUTS 3 regions; **(B)** population aged 65–79 years. Line chart showing the trend in the percentage of the population aged 65–79 years in the EU (in green) and Italy (in blue) from 2001 to 2022; **(C)** median age trends (2001–2020). Scatter plot displaying the median age trend in Europe, with data points for 2001, 2010, and 2020. The EU average is marked in orange, with individual countries represented by blue dots. Data source: Eurostat.

Heart transplantation remains the gold standard treatment for patients with end-stage heart failure, offering a life-saving option when medical and device-based therapies are no longer effective. However, strict eligibility criteria, particularly those based on age, have historically limited access to transplantation for older patients. As life expectancy increases and medical advancements improve outcomes in elderly populations, the question arises: should advanced age alone be a contraindication for heart transplantation? (Figures 1A-C).

In recent years, an increasing number of studies have explored the feasibility of heart transplantation in older adults, suggesting that well-selected elderly patients can achieve survival rates comparable to those of younger recipients. Despite concerns about higher surgical risks, immunosuppression tolerance, and long-term complications, evidence indicates that chronological age should not be the sole determinant of transplant eligibility. Instead, comprehensive patient assessments, including functional status, comorbidities, and physiological reserve, may provide a more accurate basis for candidate selection.

This case represents the oldest documented recipient discharged home in good health after transplantation, based on data provided by the Italian National Transplant Center (Centro Nazionale Trapianti - CNT), the United Network for Organ Sharing (UNOS), and Eurotransplant. By detailing the preoperative evaluation, surgical approach, and post-transplant recovery, this report aims to challenge traditional age-based criteria and highlight the potential benefits of expanding transplant eligibility for elderly patients. The findings contribute to the ongoing debate on optimizing organ allocation policies to meet the increasing demand for heart transplants in an aging population.

## Detailed case description

A 76-year-old male presented to our transplant center in February 2024 with advanced heart failure due to idiopathic dilated cardiomyopathy and severe biventricular systolic dysfunction. He had a history of hypertension, moderate chronic renal insufficiency, and paroxysmal atrial fibrillation. Since 2021, the patient had been on optimized medical therapy and had received a dual-chamber cardioverter defibrillator (ICD) for primary prevention. Despite atrioventricular node ablation with cardiac resynchronization therapy (CRT-D) in February 2024, despite optimized medical therapy (Table 1), he experienced recurrent hospital admissions for worsening congestive heart failure.

**Table 1:** Summary of optimized heart failure medical therapy prior to transplantation. This includes the core pharmacological classes recommended in current heart failure guidelines, with doses and clinical rationale tailored to the patient's comorbidities and tolerability.

Drug class	Medication	Daily dose	Comments
Beta-blocker	Bisoprolol	5 mg	Optimized to control resting heart rate
ARNI (ACEi/ARB alternative)	Sacubitril/Valsartan	49/51 mg twice daily	Well tolerated; titrated gradually
Mineralocorticoid receptor antagonist (MRA)	Eplerenone	25 mg	Renal function monitored closely
SGLT2 inhibitor	Dapagliflozin	10 mg	Initiated in 2023; tolerated without side effects
Loop diuretic	Furosemide	150 mg	For volume overload and symptom control
Anticoagulant (NOAC)	Apixaban	5 mg twice daily	For paroxysmal atrial fibrillation

**Figure 2:**
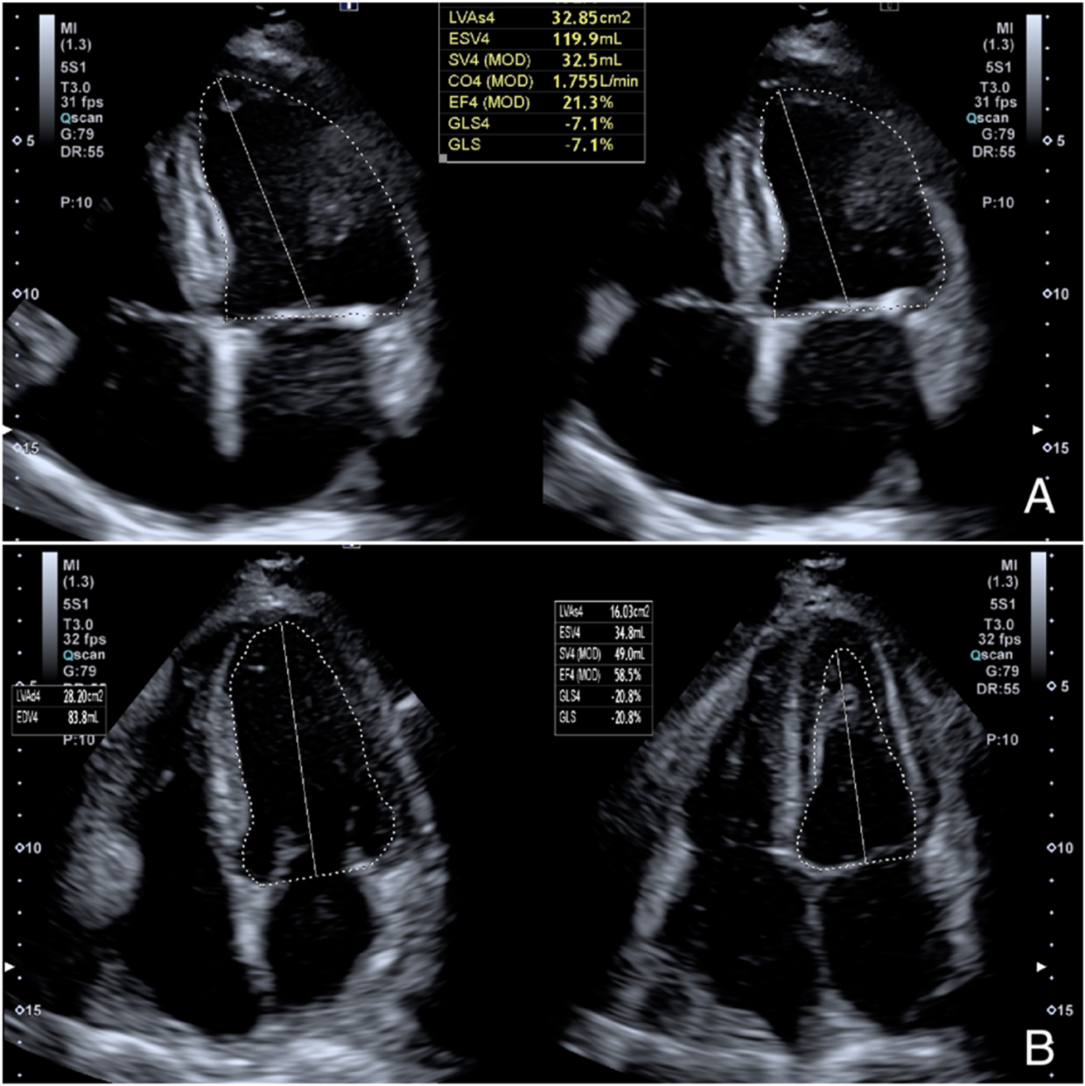
Echocardiogram (Toshiba Aplio i700 ultrasound scanner) showing in apical 4-chamber projection an LVEF of 21% calculated by Simpson's monoplane method **(A)**; eight months after transplantation, the echocardiogram shows normal systolic function with an LVEF of 58% and a GLS of −20.8% **(B)** LVEF, left ventricular ejection fraction; GLS, global longitudinal strain.

After diuretic therapy, right heart catheterization indicated mildly elevated systolic pulmonary artery pressure (PAPs 30 mmHg, PAPm 15 mmHg, PCWP 12 mmHg), along with a reduced cardiac index (1.6 L/min/m^2^) and cardiac output (3.2 L/min). The patient underwent a cardiopulmonary exercise test (CPET) which showed a peak VO₂ of 11. 5 ml/kg/min, corresponding to 35% of the predicted value, and a VE/VCO₂ slope of 35. Transthoracic echocardiography showed increased heart chamber size with severe systolic dysfunction, with a left ventricular ejection fraction (LVEF) of 20%–25% and a global longitudinal strain (GLS) of −7% (Figure 2A). A comprehensive preoperative evaluation was essential to determine the patient's suitability for heart transplantation. Multiple validated risk scores were employed to assess mortality risk, hepatorenal function, and overall surgical candidacy. As summarized in Table 2, the patient presented with a MECKI score of 36%, indicating a moderate-to-high 2-year mortality risk with medical therapy alone, and an IMPACT score of 7, corresponding to intermediate post-transplant risk. Additional assessments, including a MELD-XI score of 13 and a HeartMate II Risk Score of 1.2, further supported the decision to proceed with transplantation rather than left ventricular assist device (LVAD) therapy. The Clinical Frailty Scale score of 3 confirmed adequate functional reserve and absence of frailty, reinforcing the patient's eligibility. After completing the screening, he was subsequently listed for cardiac transplantation in march 2024.

**Table 2:** Comprehensive preoperative risk assessment scores for the 76-year-old heart transplant candidate. These scores were used to estimate mortality risk, evaluate hepatorenal function, and determine surgical candidacy.

Score	Patient value	Interpretation
MECKI score	36%	Estimated 2-year mortality with optimal medical therapy: moderate-to-high risk.
IMPACT score	7	Intermediate post-transplant mortality risk.
MELD-XI score	13	Moderate hepatorenal dysfunction; supports transplant over LVAD.
HeartMate II risk score	1.2	Intermediate surgical risk if LVAD were considered.
Clinical frailty scale	3 (managing well)	Non-frail; good functional reserve, appropriate for transplant.

A suitable 66-year-old female donor became available several weeks later. The donor, who was in brain death due to cranial trauma, had an unremarkable cardiac history. Electrocardiography demonstrated sinus rhythm at 75 bpm. Echocardiography revealed normal biventricular function and no significant valvular disease. Coronary angiography showed no hemodynamically significant epicardial stenoses. Both virtual and crossmatch testing were negative.

Orthotopic heart transplantation was performed using the standard bicaval technique, with an ischemic time of 3 h and 40 min. At the end of the procedure, the patient was transferred to the ICU. Prophylactic antibiotic therapy was started with Piperacillin/Tazobactam 4.5 g/0.5 g QID and Vancomycin 500 mg daily for 3 days. He received anti-thymocyte immunoglobulins 25 mg for 4 days and methylprednisolone 1 g after aortic declamping and then 125 mg TID for 2 days for induction immunosuppression. Maintaining therapy included corticosteroids (prednisone 25 mg) and tacrolimus, whose serum levels were kept at the lowest efficient level (7–10 ng/ml). On the fifteenth day after the transplant, mycophenolate mofetil was introduced into the therapy at a dose of 500 mg twice daily. The postoperative course was uncomplicated. Extubation occurred on postoperative day 1, and transfer to the recovery ward on postoperative day 3. The patient showed an excellent recovery. Post-transplant echocardiography controls demonstrated normal biventricular function, and a 15-day biopsy showed no evidence of rejection (ISHLT 0R, 2004 grading). The patient was discharged on postoperative day 30. At the 14-month follow-up, the patient continued to be in good health and exhibited excellent biventricular function, with an LVEF of 58% and a GLS of −20.8% on echocardiogram (Figure 2B). The 6-minute walk test (6MWT) increased from 180 meters pre-transplant to 405 m at follow-up. A cardiopulmonary exercise test (CPET) performed at 12 months demonstrated a peak VO₂ of 17.2 ml/kg/min, indicating preserved functional capacity. The patient reported a KCCQ (Kansas City Cardiomyopathy Questionnaire) overall summary score of 86/100, up from 32/100 at baseline, reflecting a marked improvement in symptom burden and daily functioning. On post-transplant reassessment, frailty status had improved (Clinical Frailty Scale score: from 3 to 2), and the patient resumed light recreational physical activities and reported improved psychosocial well-being.

## Discussion

Heart transplantation remains the gold standard treatment for end-stage heart failure (1). However, donor shortages continue to challenge the transplant community, with demand exceeding supply (2). At the same time, an increasing number of elderly patients are being considered for transplantation (3).

As of January 1, 2023, the median age in EU countries ranged from 38.4 years (Cyprus) to 48.4 years (Italy). Over the past decade, the median age increased in most EU countries, with Italy among those registering a 4-year rise (4). Advancements in medical therapy have enabled more older patients to qualify for transplant lists (5). However, age-related comorbidities make heart transplantation increasingly complex (2).

Italy is experiencing significant demographic shifts, characterized by a declining birthrate and an aging population. In 2023, the country recorded only 379,000 births, the lowest number since its unification in 1861. This decline is attributed to factors such as economic instability, housing challenges, and delayed family planning. Consequently, nearly 24.1% of Italians are now over the age of 65, making Italy one of the oldest countries globally.

This demographic trend poses challenges for the nation's economy and social services, particularly in healthcare systems. The increasing old-age dependency ratio places additional pressure on public finances, necessitating reforms in retirement policies and healthcare provision (4).

Similarly, Europe as a whole is grappling with an aging population. The European Union recorded 3.665 million births in 2023, the lowest since 1961, highlighting a continent-wide demographic challenge. Factors contributing to this trend include economic uncertainties, shifting societal values, and increased life expectancy (4).

In summary, both Italy and Europe are facing significant demographic shifts due to declining birthrates and aging populations. Addressing these challenges requires multifaceted approaches, including policy reforms, economic incentives, and societal changes to ensure sustainable futures.

In our center, the average age of transplant candidates has risen from 55.7 to 62.3 years over the past five years. In the last two years, fourteen patients over seventy have successfully undergone transplantation.

Recent studies have explored the impact of an aging population on heart transplantation outcomes. Traditionally, advanced age was considered a contraindication for heart transplantation due to concerns about increased morbidity and mortality. However, emerging evidence suggests that selected elderly patients can experience favorable outcomes post-transplant.

A retrospective analysis of the U.S. Scientific Registry of Transplant Recipients from 2006 to 2022 examined outcomes in heart transplant recipients aged 70 and older. The study found that these patients had a one-year survival rate of 87.5%, compared to 91.1% for those under 60 and 88.4% for those aged 60–69. While the difference was statistically significant (*p* < 0.001), the survival rate for septuagenarians remained high, indicating that heart transplantation can be a viable option for carefully selected elderly patients (6).

Another study utilizing data from the United Network for Organ Sharing (UNOS) database analyzed heart transplant recipients from 1987 to 2014, stratifying them by age groups: 18–59, 60–69, and 70 and above. The findings revealed that five-year mortality rates were 26.9% for recipients aged 18–59, 29.3% for those aged 60–69, and 30.8% for recipients aged 70 and above. Despite advanced age and the use of older donors, recipients in their 70s had outcomes comparable to those in their 60s, suggesting that selected older patients should not be routinely excluded from consideration for heart transplantation (7).

These studies highlight the importance of individualized patient assessment over chronological age alone when determining eligibility for heart transplantation. As the population continues to age, expanding transplant criteria to include well-selected elderly patients could help address the growing demand for heart transplants.

This case highlights the importance of integrating geriatric principles into transplant evaluation protocols, particularly in an aging population. A comprehensive preoperative assessment included evaluation of comorbidities using the Charlson Comorbidity Index, which confirmed a moderate burden of chronic illness. Functional status was also assessed, and the patient was deemed non-frail based on the Clinical Frailty Scale (score of 3), supporting his candidacy despite chronological age. Multidisciplinary decision-making played a central role in this case. The transplant team collaborated closely with specialists in geriatrics, cardiology, nephrology, and anesthesiology to conduct a holistic risk-benefit evaluation. Such interdisciplinary coordination is essential in elderly patients, whose outcomes may depend as much on physiological reserve and psychosocial resilience as on cardiac function alone. Given the growing demand for transplants among older adults, this case supports the incorporation of geriatric-specific screening protocols—including frailty indices, cognitive assessments, and social support evaluations—into routine transplant candidacy workflows. Doing so could enhance candidate selection, optimize resource use, and promote more equitable access for older patients with favorable biological profiles.

This case also raises important ethical considerations in the allocation of scarce donor organs. The decision to allocate a donor heart to a 76-year-old recipient was made following a comprehensive, multidisciplinary assessment and in alignment with national and institutional transplant policies. Ethical justification was grounded in the principle of equity, whereby access to life-saving therapy is based on individual clinical merit rather than chronological age alone. The recipient demonstrated a non-frail physiological profile, moderate comorbidity burden, and strong psychosocial support—all key predictors of favorable post-transplant outcomes. Italy does not impose strict age-based exclusion criteria for transplantation. Instead, organ allocation follows the Italian National Transplant Center (CNT) guidelines, which emphasize medical urgency, compatibility, and anticipated benefit. In this case, the donor heart—a 66-year-old organ—was deemed less suitable for younger recipients with longer expected lifespans, but well-matched to an elderly recipient with similar biological age, thus respecting the principle of “age-matched” allocation to optimize utility without compromising fairness. Balancing utility (maximizing the benefit from a limited resource) with justice (ensuring fair access) remains central to organ allocation. This case supports a nuanced approach that incorporates biological age, frailty, and potential for recovery, rather than excluding elderly candidates *a priori*. Such cases challenge traditional allocation models and highlight the need for adaptive, individualized criteria in the context of demographic shifts and increased longevity.

The growing aging population worldwide necessitates a reevaluation of transplant eligibility criteria. As donor shortages persist, expanding the recipient pool to include well-selected older candidates could help bridge the gap between organ availability and demand. Recent studies have reinforced the idea that age alone is not the primary determinant of post-transplant survival, with factors such as overall health status, absence of significant comorbidities, and functional capacity playing a more critical role.

## Conclusions

This case documents the successful heart transplantation of a 76-year-old patient, marking the oldest known recipient discharged home in good health based on data from the Centro Nazionale Trapianti, UNOS, and Eurotransplant. The outcome challenges the use of strict chronological age as a limiting criterion for heart transplant eligibility. Our experience reinforces the importance of individualized patient selection based on biological age, functional capacity, and comorbidity profile. With appropriate screening and multidisciplinary assessment, well-selected elderly patients can achieve excellent post-transplant outcomes. As populations age and donor shortages persist, expanding eligibility criteria to include non-frail older adults may help bridge the gap between organ supply and demand. This case supports a shift toward personalized, physiology-based decision-making in transplant medicine, opening new frontiers in equitable access to life-saving therapy.

## Data Availability

The original contributions presented in the study are included in the article/Supplementary Material, further inquiries can be directed to the corresponding author.
